# Assessing the Processes and Experiences of Using and Implementing a Routine Data Collection System at Two Aboriginal Alcohol and Other Drug Residential Services Located in Rural Queensland

**DOI:** 10.1111/dar.14095

**Published:** 2025-06-08

**Authors:** Katinka van de Ven, Erin Cunningham, Phillip J. Tully, Alison Ritter

**Affiliations:** ^1^ 360Edge Melbourne Australia; ^2^ Centre for Rural Criminology, School of Humanities, Arts, and Social Sciences University of New England Armidale Australia; ^3^ Drug Policy Modelling Program Social Policy Research Centre, UNSW Sydney Sydney Australia; ^4^ Cape York Family Centre Cooktown Australia; ^5^ Stagpole Street Drug and Alcohol Rehabilitation Unit Townsville Australia; ^6^ School of Psychology Deakin University Geelong Australia; ^7^ School of Medicine The University of Adelaide Adelaide Australia

**Keywords:** Aboriginal health, alcohol and other drugs, client outcomes, participatory evaluation

## Abstract

**Introduction:**

Routine data collection in alcohol and other drug (AOD) treatment services is essential for continuous quality improvement, yet its implementation in Aboriginal residential services remains challenging. This study evaluates the processes and experiences of implementing a routine data collection system at two Aboriginal AOD residential rehabilitation services in rural Queensland, both of which operate under a family‐centric model of care.

**Methods:**

A participatory evaluation approach was used, engaging staff and clients to co‐design and implement data collection tools. The study progressed in two phases. Phase 1 focused on developing data collection tools. Phase 2 involved implementing these tools to assess client experiences and outcomes. Data collection included the Aboriginal Resilience and Recovery Questionnaire, WHOQOL‐BREF and other validated AOD outcome measures, with surveys conducted at multiple time points.

**Results:**

Staff engagement was crucial to successful implementation, though maintaining involvement over time proved challenging. Logistical and financial barriers, including IT infrastructure limitations and staff turnover, impacted sustainability. Although post‐treatment follow‐up was difficult, many clients found the outcome survey to be a valuable therapeutic tool. The client experience survey led to direct service improvements, whereas outcome monitoring required additional refinement for long‐term feasibility.

**Discussion and Conclusions:**

Routine data collection can enhance service quality and client outcomes when embedded into clinical practice. However, sustainability depends on staff buy‐in, streamlined processes and dedicated resources. Addressing barriers to post‐treatment follow‐up and ensuring data collection remains relevant to both staff and clients will be essential for ongoing implementation.

## 1 Introduction

Aboriginal and Torres Strait Islander peoples have maintained complex knowledges, cultures and spiritual connections to Australian lands and seas for more than 65,000 years; however, they have suffered because of colonisation, dispossession, marginalisation, racism and structural inequities. The longstanding impacts are reflected in alcohol and other drug (AOD) related morbidity, which is disproportionately high among this population [1]. Risky AOD use can be a concern for Aboriginal and Torres Strait Islander peoples and communities in Australia because it may impact a person's cultural, kinship and spiritual connections. In addition to the universal challenges that arise from AOD use, there are specific concerns related to cultural factors that impact risky use within Aboriginal communities, such as the impact of colonisation on risky drinking [2].

In 2022–2023, Aboriginal and Torres Strait Islander clients accounted for 18% of all clients treated by publicly funded AOD treatment services in Australia [3]. Culture impacts Aboriginal and Torres Strait Islander peoples' experiences of AOD treatment, with self‐determination and cultural connection being important [4]. Although Indigenous healing practices and cultural values are increasingly incorporated in treatment services, most AOD treatment and support in Australia and internationally is based on Western principles of health and well‐being [5]. It tends to be individualised, with the problem and solution being located within the person using the substance. Treatment interventions are generally provided for individuals, away from their families. For Aboriginal and Torres Strait Islander peoples, health and well‐being are not just about the individual. They are understood through a holistic context that focuses on the physical, spiritual, cultural, emotional and social well‐being of the individual, family and community. The involvement of family in the treatment for AOD use has been identified as a key priority for Aboriginal and Torres Strait Islander communities in their recovery journey [6, 7, 8]. Research shows that non‐Indigenous people also benefit from a family‐centric approach to therapy [9]. Family and community can play an essential role in treatment initiation, reducing or ceasing AOD use and avoiding relapse [9, 10, 11, 12].

Although AOD treatment services in Australia are increasingly adopting family‐focused interventions, this is still limited [9]. Family members and the community are often not involved in the treatment process of the client [10]. Despite there being many promising treatment approaches reported for this population, little research exists that evaluates the effectiveness of a family‐centric model of care on client outcomes for Aboriginal and Torres Strait Islander peoples who experience AOD issues, with a limited number of outcome evaluation studies [13, 14, 15, 16, 17]. In addition, little research exists on understanding implementation barriers and enablers of ongoing data collection and monitoring in Aboriginal AOD services.

One way to strengthen effectiveness research in this area is to embed routine data collection within Aboriginal and Torres Strait Islander treatment services as part of their ongoing practice and to support quality improvement initiatives. The study reported here evaluates the implementation of routine outcome data collection within two Aboriginal and Torres Strait Islander AOD residential rehabilitation treatment services located in rural Queensland (Australia) that offer family‐centric models of care. Quality improvement involves gathering empirical and data‐driven information about healthcare to improve client outcomes over time [18]. Quality improvement initiatives in healthcare are varied and may focus on systems, processes, technologies, organisational factors and/or the quality of data capture itself.

The study aimed to better understand and improve the ways in which Pinangba's two Aboriginal and Torres Strait Islander AOD residential rehabilitation treatment services routinely collect and collate clinical data to build a culture of ongoing service improvement and evaluation within their services. For this paper, we particularly focus on the development and implementation of an ongoing data collection system at Pinangba and the barriers and enablers to routine data monitoring and evaluation becoming a standard process.

## 2 Methods

### 2.1 Setting

Pinangba is an Aboriginal and Torres Strait Islander‐led provider of AOD treatment. Most clients identify as Aboriginal and/or Torres Strait Islander (92.3%). Most staff, including management, identify as Aboriginal and/or Torres Strait Islander, including the manager who led this project (E.C.). Pinangba adopts a systematic family therapy model of care [19, 20] and allows for the entire family unit to undertake treatment. Two of Pinangba's residential rehabilitation services were included in this study.

### 2.2 Design

The study employed a participatory evaluation design [21], which involved engaging clients and staff as key stakeholders to foster Aboriginal and Torres Strait Islander leadership, establish collaborative partnerships and empower the two Pinangba services to shape the research focus, process, findings and implications. The study progressed in two phases. Phase 1 entailed developing and designing data collection tools to be implemented in Phase 2. Three data collection methods were used in Phase 1: an analysis of administrative client data from 2017 to 2020, semi‐structured interviews with clients and staff and workshops with clients and staff piloting the newly developed survey to measure outcomes. This process aimed to conclude with a routine data collection system reflecting the input of all stakeholders, with two survey instruments: experience and outcome. It also aimed to determine optimal data collection methods and timing, and how to implement it within the service.

Phase 2 focused on implementing routine data collection at both services, including incorporating the survey instruments to measure client outcomes. Regular meetings (weekly in Phase 1 and monthly in Phase 2) were held with the project lead (E.C.) and external researcher (K.V.) to better understand the barriers and enablers of implementing routine monitoring and evaluation into the services. The project lead also regularly met with her senior manager and other staff members to reflect on the outcome monitoring process. The project lead kept a record of all encountered issues. She also kept notes of reflections from clients on the process when conducting the survey instruments with the clients.

This article reports on the outcomes of Phase 2, which focused on assessing the processes and experiences of implementing a routine data collection system at Pinangba's two Aboriginal AOD residential services.

### 2.3 Routine Data Collection Tools

#### 2.3.1 Client Experience Survey

The validated Patient Reported Experience Measure for Addiction Treatment (PREMAT) tool [22] was used to assess client experience. The client experience survey is a 25‐item measure which captures the experience of clients accessing Pinangba residential AOD treatment, including emotional well‐being, personal privacy, daily structure, relationships with staff and peers, access to support for health, financial, and legal issues and preparation for life after the program. It also assesses clients' perceptions of program rules, opportunities for personal growth, and access to information and external services. It includes 23 statements scored using a Likert scale (from ‘*strongly disagree*’ to ‘*strongly agree*’) and two open‐ended questions. Some small wording adjustments were made to ensure the language used aligned with the service; the developers of the PREMAT were consulted to ensure validity was not impacted.

#### 2.3.2 Client Outcome Survey

The client outcome survey consists of six sections. Section 1 collects demographic data, including marital status, living arrangements, education, employment, legal circumstances, referral type, mental health diagnosis, medication use and Aboriginal and/or Torres Strait Islander status. Section 2 assesses resilience, healing and recovery using the validated 19‐item Aboriginal Resilience and Recovery Questionnaire (ARRQ), which measures cultural, relationship and personal strengths on a 5‐point Likert scale. This section is skipped for non‐Indigenous clients.

Section 3 evaluates AOD use using validated tools, including the Severity of Dependence Scale, the Australian Alcohol Treatment Outcome Measure and the Brief Treatment Outcome Measure‐Concise, capturing the primary substance of concern, usage patterns and quantity. Section 4 measures clients' confidence and capacity to manage life aspects such as housing and employment through a 10‐item scale developed in consultation with clients, families, staff and advisory groups. Section 5 assesses quality of life using the WHOQOL‐BREF, a 26‐item tool measuring physical and psychological health, social relationships and environmental factors. Finally, Section 6 provides an open text box for client feedback. More detail about the client outcome survey is provided in the Supporting Information.

### 2.4 Procedure

We outline the procedure of conducting the experience and outcome surveys below, as the way it was implemented impacted the ability of the project lead and Pinangba staff to collect and manage the data collection and analysis at both services.

#### 2.4.1 Client Experience Survey

The survey was conducted three times over a 12‐month period between September 2022 and September 2023, each time being open for approximately 3 weeks. The 3‐week period was selected based on input from clients and staff regarding when they ideally would like the survey to be implemented and how long data collection should take place. All clients who were in treatment at that time were approached, regardless of how many weeks they had been in treatment. Clients were informed at general client meetings about the option to fill out an anonymous survey either in their own time or during client group meetings. Clients could fill out a hard copy and submit it in an enclosed box or fill it out and submit it via a survey software platform on a computer or iPad. Due to the length of the program, clients had generally completed or left the service before all three timepoints, and the survey was filled out by different clients at each timepoint. The second author (E.C.) collected all the data and, together with the first author (K.V.) analysed it and summarised it. The data were stored in the same survey software program in which the data were collected (Qualtrics). As part of the goal to integrate research into practice, the outcomes of the experience survey have been reported to the manager and via staff and client meetings.

#### 2.4.2 Client Outcome Survey

The administrative officers, not directly involved in delivery of care, approached clients within the first 2 weeks of treatment entry inviting them to participate. Informed consent was obtained and participants were provided with a payment of AU$30.00 at the end of each interview as compensation for their time. After consent, the project lead at Pinangba (E.C.) then scheduled the interviews in consultation with the case manager of the client. To assist the researcher with locating clients for the post‐treatment interviews, participants were asked to provide names and contact details of people who are close to them in a locator form.

The client outcome survey was conducted on five occasions: (i) first 2 weeks of admission; (ii) 2 months post‐admission; (iii) 3 weeks before discharge; (iv) 1 month post discharge; and (v) 3 months post discharge. All sections were completed at all timepoints, except for the section regarding their AOD use, which was not collected during inpatient treatment (Timepoint 2) as the assumption was that clients were abstinent. Surveys were completed in person, online (Zoom) and via phone.

The second author (E.C.) filled out the client outcome survey with the client, either electronically (directly into the survey software program) or via paper and pen. If filled out using paper or pen, the data were directly inserted into the survey software program to ensure it was all stored in the same place. The second author collected all the data, and together with the first author (K.V.), analysed and summarised it. The data were stored in the same survey software program in which the data were collected (Qualtrics). Outcomes of the outcome survey have been reported to the manager, the case managers of the client (with permission of the client) and individual clients.

### 2.5 Analysis

An interpretative thematic analysis approach was undertaken for the qualitative data, using an iterative, inductive process whereby the project lead (E.C.) and external researcher (K.V.) read the notes to identify recurring themes and patterns of meaning and reflected on salient domains and issues [23]. A list of the encountered barriers and enablers was generated and grouped into themes. Although this study was not an evaluation of the services, we do report the experience and outcome data that were obtained using descriptive statistics, reporting ‘*agree*’ and ‘*strongly agree*’ responses for the experience survey and descriptive statistics across the outcome domains for the outcome survey. These are reported in the Supporting Information.

### 2.6 Ethics Approval

The ethical aspects of this research project have been approved by the UnitingCare Human Research Ethics Committee (UCQ HREC; approval no. 08102020) and the Australian Institute of Aboriginal and Torres Strait Islander Studies (AIATSIS) Research Ethics Committee (approval no. EO202‐20200929). Ethics approval was also obtained from the Victorian Aboriginal Health Service (VAHS) Ethics Committee to use the ARRQ as part of the routine data collection protocol.

## 3 Results

### 3.1 The Importance of Staff Buy‐In

Several steps were undertaken to ensure staff were involved and invested in the project, particularly during Phase 1. We worked closely with staff from all levels, including senior managers, case managers and administrative officers. Staff members were involved in the development and implementation of the ongoing data collection system in Phase 1. Meetings and training sessions with all staff members had also been organised throughout Phase 1 around a range of aspects, such as obtaining consent and the importance of data collection. Feedback from staff members in Phase 1 indicated that they appreciated being involved in the project, felt heard and understood the value of data collection.

Nevertheless, it was hard to get staff members involved and invested in recruitment and data collection for the outcome survey in Phase 2. First, administrative officers found it difficult to build the project requirements into their daily routines. The recruitment of clients to the project was considered an added responsibility to their already busy agendas. Administrative staff felt that they did not have enough time to approach clients and that it risked other responsibilities being neglected. As a result, clients were not always approached in time, or when approached, administrative officers were quick in their explanations on what the project was about. A meeting was planned with the senior manager of both services to discuss this issue, who ensured that the administrative officers would be provided with more support, such as having the phone and desk manned by someone else while they conducted recruitment. The senior manager also expressed to the administrative officers that it was understood that these added responsibilities could impact other duties. This seemed to improve client recruitment. Once the project element is gone and no consent is required, conducting the client outcome survey will become a standard part of both services and will be added to the checklist of activities that a client needs to complete during the different program phases.

Second, staff also found it hard to stay engaged with the project due to the data collection process for the outcome survey being long, taking approximately 2 years. Outcomes for individual clients were not communicated throughout the project due to ethical constraints and group level data could not be provided until the end of the project. Pinangba staff were not very engaged as they perceived the data to not be used in a direct, purposeful manner. However, when the project lead provided direct feedback to a case manager, they became more engaged; for example, discussing areas the client wanted to work on.

Case managers also indicated that they did not understand the aim of the project and were less inclined to support the project for this reason. They also did not always have a clear understanding of their role, with some trying to recruit clients although this was the responsibility of the administrative officer. Meetings with case managers and other staff members were regularly planned in Phase 1, which involved setting up the project and collaborating with staff members in the development and implementation of the routine data collection system. This did not continue in Phase 2.

Building the capacity of all Pinangba staff to put the routine data collection system in place for Phase 2 to commence was a central aspect. Phase 2 was about testing the ongoing data collection system and collecting data and so was more focused on building the capacity of the project lead (E.C.) to conduct routine data collection at Pinangba. Informal meetings were organised on an ad hoc basis during this time with individual case managers and other staff members. Reflecting on this issue, we should have built in regular engagement opportunities in Phase 2 to keep all staff members informed about the progress of the project and to refresh their memories, particularly since staff left and new ones were hired during this time (see below).

However, staff were highly engaged with the experience survey, as data collection and analysis were considered efficient processes. Data for the survey were collected in approximately 3 weeks and were communicated in a timely fashion approximately 4–6 weeks after data collection had ended, with changes to service provision being made based on the outcomes. In addition, as this was an anonymous survey, no consent needed to be asked, avoiding the additional layer of needing to involve staff members in recruitment. There was also little additional burden placed on case managers as clients filled the survey out in their own time and the researchers analysed the data. The burden on clients and the project lead was also low as the survey was short. As a result, data analysis was relatively quick and simple.

### 3.2 Issues With Data Collection and Management

The costs, IT infrastructure needed, and expertise required to collect and analyse the data were barriers to ensuring the outcome monitoring process became a standard process at the services. The project team initially examined whether internal systems were in place through which the data could be collected and analysed. Although some programs were available, these were managed by the larger organisation Blue Care and meant that Pinangba would not have direct access to the survey, data and outputs. There were also limitations around what could be included in the survey and what outputs could be generated and when. Survey platforms and analysis software are costly and something for which AOD services generally are not funded Although Pinangba was very supportive and willing to purchase the programs needed, we were able to make the project lead (E.C.) an Honorary Associate at the University of New England. As a result, she has access to the programs needed to collect and analyse the data. However, this Honorary Associate role is for 3 years and only provides individual access to a range of software.

In addition, the technical skills required to understand the data and analyse whether it is relatively complex, particularly when being completely new to it. Although building the research capacity of the project lead was a central aspect of this project, this was hindered at times by other work responsibilities taking priority, such as taking over responsibilities of staff members who leave, and personal and medical issues. Nevertheless, the project lead completed courses and training sessions on data management and analysis. There is also an issue that these new skills are not constantly applied, resulting in the project lead not feeling confident that she will retain what she has learned (although extensive guidelines have been created). Regardless, the project lead has successfully completed and analysed the two surveys with support from the external researchers. Although this has been a huge achievement, a risk is that the outcome monitoring system becomes dependent on one person. Systems will need to be put in place to ensure work can be progressed even if the project lead were to leave Pinangba, such as training other staff members.

There were also several issues that impacted the collection of data. First, children were present at some of the online interviews with clients of the Cookstown service. At this service, the entire family unit is taken in, including children. At the beginning of the project, this was not taken into consideration and no arrangements were made for the children when an interview was scheduled. Having children present during the interview makes it hard for the client to engage. After experiencing this issue, the project lead made sure that childcare support was arranged during the time of the interview.

Second, as a research project, consent needed to be obtained for the outcome survey by someone who was not involved in the therapeutic process of the client or the research (to reduce the risk of coercion). As a result, this was conducted by the administrative officers. The lack of buy‐in from this group has been discussed above, but due to this additional step, there was also the issue that clients saw the project as something external to their treatment journey. They felt overwhelmed by the consent process and as a result were less willing to participate. Once the project has ended, filling out the outcome survey will become a standard ongoing process completed by every client at different time points, with consent no longer being required.

Finally, some challenges were experienced with the survey itself. Although the ongoing data collection system was piloted in Phase 1, clients did experience some difficulties with answering all the questions of the outcome survey. The project lead noted that some clients did not have the intellectual capacity to respond to some of the abstract questions. Clients also felt that some questions were ambiguous and found them confusing. The project lead therefore had to interpret some of the questions in different ways depending on the client, which may have impacted the validity of the instrument. Instructions will need to be set up for these questions to ensure they are explained in a similar manner if this issue arises. In addition, the project lead noted that the outcome survey is overly long. Clients also indicated that they found it hard to retain their attention. Furthermore, the project lead noted that the analysis for the client outcome survey was quite complex and time consuming, particularly the scoring of the WHOQOL‐BREF. Some adjustments will therefore need to be made to the outcome survey to ensure the project lead can continue to manage it as part of her daily responsibilities. For example, certain survey sections that are already collected, like demographics, are not necessary and there is some doubling up of items such as physical health.

### 3.3 Post‐Treatment Follow‐Up

A barrier to implementation was that most clients did not complete the follow‐up surveys. The client outcome survey was administered from September 2022 to November 2023. One hundred and eleven clients entered treatment during this time. In total, 71 clients (64.0%) consented to participate in the project. Of the 71 clients, 26 (36.6%) filled out an admission survey, 12 (16.9%) filled out a 2‐month survey (in treatment) and 10 (14.1%) filled out a discharge survey. Only one person filled out a post‐treatment survey.

Despite the project lead feeling that she had built a strong rapport with the clients of both services and having a locator form, most could not be located or were no longer interested in participating once they left. It did not matter whether clients successfully graduated from the program or not. Only one client filled a 1‐month post‐treatment survey, and no 3‐month post‐treatment surveys were completed.

The project lead noted that it was harder to build rapport online than face‐to‐face with clients. She is stationed in Townsville and all client engagement could therefore be conducted in person at the Townsville service. Although the project lead visited the Cookstown service several times throughout the project, this was not always possible because of work commitments, personal issues and/or external factors such as flooding. Furthermore, with clients regularly entering and leaving the Cookstown service, it was often not feasible to arrange a face‐to‐face meeting in time. Appointments to conduct the survey therefore often took place online. To solve this issue, a staff member located at the Cookstown service was approached to support the project lead, but this was not until the end of data collection.

### 3.4 Staff Turnover

During the time of data collection, both services had difficulties retaining qualified and experienced staff, with the Townsville service particularly being impacted by staff leaving. This was because a performance review of this service was conducted during the project, which resulted in a restructuring of staff. Throughout the project, several administrative officers left, and the process of hiring new administrative officers and training them on how to recruit clients slowed the client recruitment process down. Data collection was also hindered because of case managers leaving. Ensuring the project lead was informed about the treatment progression of clients was sometimes hindered because of clients being handed over to new case managers. For example, the new case manager was not always made aware that the client was participating in the project, resulting in the project lead not being informed when a client discharged. Little could be done to prevent staff leaving or to speed up the recruitment process, but more communication mechanisms should have been built into Phase 2 to reduce the impact of staff turnover on data recruitment and collection.

### 3.5 Outcome Monitoring as a Therapeutic Process

An unexpected outcome of engaging clients in this quality improvement process was that most indicated that filling out the survey felt like a therapeutic intervention. It led clients to reflect on aspects of their healing journey they had not considered before. At the end of completing the survey with the project lead, clients often reflected on what aspects particularly mattered to them and whether this could be disclosed to their case manager for inclusion in their treatment plan. For example, one client realised that they did not know where their families' cultural connection came from or the country they were connected to. The client included this as part of their recovery journey and was able to locate from which country their family was from before discharging from the program.

Clients also used the interviews as an opportunity to reflect on strengths and challenges of the services they received. For example, a client disclosed that they wished to have more regular counselling sessions. Permission was obtained from the client to discuss this with their case worker and more sessions were arranged with the client as part of their treatment plan. In relation to information sharing, the second author found it hard to navigate this line during the project as to what should remain confidential or not. An example is when a client disclosed that they drank or used other drugs on site (unprompted by the outcome survey). This could result in a client being discharged and can cause an unsafe environment if not disclosed to the case manager and addressed. For the project, no information was shared with other staff members unless a client consented to this.

## 4 Discussion

The implementation of a routine data collection system at Pinangba revealed critical insights into both the benefits and challenges of embedding outcome monitoring and evaluation into practice. The findings underscore that although routine data collection can enhance service delivery and client outcomes, its success is contingent on staff engagement, adequate resourcing, and the adaptability of tools to the cultural and practical needs of the service.

A key enabler of successful data collection was the participatory approach used, which fostered engagement among staff and clients. Staff involvement in the development and implementation of data collection tools during Phase 1 resulted in increased buy‐in. Research shows that staff engagement is essential for the success of routine outcome monitoring in AOD services [24, 25]. However, as seen in Phase 2, engagement waned over time, particularly among administrative officers and case managers, due to competing work responsibilities and a lack of direct, real‐time application of collected data. This aligns with broader research findings that staff disengagement can arise when outcome monitoring is perceived as an administrative burden rather than a clinical tool [25, 26]. Future implementations should ensure continuous feedback loops to demonstrate the immediate value of data collection in client care and service improvement.

The study also highlighted significant logistical and structural barriers to data collection, including IT infrastructure limitations, funding constraints and the complexity of data analysis. These challenges are consistent with existing literature identifying the costs and technical demands of data management as a common issue in AOD treatment services [26, 27]. Despite external funding enabling access to survey software and statistical analysis tools, sustainability remains a concern. Without dedicated funding and internal expertise, maintaining routine data collection may become unfeasible. Pinangba may need to explore long‐term solutions such as integrating data collection into existing electronic health records or securing dedicated funding for data monitoring and evaluation.

A major challenge was post‐treatment follow‐up, with very few clients completing surveys after leaving the service. This difficulty in tracking clients post‐treatment is widely documented in AOD research, where follow‐up response rates tend to be low because of factors such as phone number changes or clients' desire to disengage from services after completing treatment [26]. Although the use of a locator form was intended to mitigate this issue, alternative strategies, such as stronger rapport‐building mechanisms and the use of digital engagement tools, should be explored to improve long‐term follow‐up rates [28].

One unexpected but positive finding was that many clients viewed the survey process as a therapeutic tool, using it as an opportunity to reflect on their progress and identify personal goals. This supports research showing that routine outcome monitoring can enhance the therapeutic process by facilitating self‐reflection, increasing client engagement and strengthening the therapeutic alliance [29, 30]. Moving forward, integrating outcome monitoring into routine clinical practice, rather than positioning it as a research activity, could further enhance its acceptability and effectiveness.

The client experience survey was particularly well received because of its efficiency in administration, analysis and reporting. Service refinements were made in direct response to survey findings such as adjustments to the client admission process. This supports existing evidence that real‐time feedback mechanisms enhance service improvement efforts [31]. Ensuring that the outcome survey is similarly streamlined, with reduced length and complexity, could further support long‐term implementation.

Several limitations should be acknowledged. First, this study was supported by research funding, which facilitated access to external expertise, software and time allocations for data collection and analysis. The feasibility of maintaining routine data collection without such funding remains uncertain. Second, although validated tools were used, some clients found certain survey questions ambiguous or challenging to answer. Refining the tools to enhance clarity and cultural relevance would improve their effectiveness. Finally, data collection for the experience survey occurred at set intervals rather than being integrated into routine clinical practice. Shifting to a model where data collection is embedded in ongoing service operations may further enhance participation and sustainability.

## 5 Conclusion

This study highlights the feasibility and challenges of implementing a routine data collection system in Aboriginal AOD residential services. Although outcome monitoring has clear benefits for service improvement and client care, long‐term success requires strong staff engagement, sustainable funding models and integration into daily practice. Addressing barriers related to staff workload, IT infrastructure and post‐treatment follow‐up will be crucial for ensuring the sustainability of these efforts. Future work should focus on refining data collection tools, increasing the therapeutic utility of outcome measures, and embedding outcome monitoring into service workflows to maximise its impact on client outcomes and service delivery.

## Author Contributions

K.V. and E.C. collected and summarized the data. K.V., E.C., P.J.T. and A.R. drafted the paper. All authors were involved in editing the final version of the paper.

## Ethics Statement

UnitingCare HREC and AIATSIS HREC are both registered with the National Health and Medical Research Council. UnitingCare HREC approval was required as Pinangba falls under UnitingCare and all research projects require approval from their own HREC. AIATSIS HREC approval was required as this research project is focused on Aboriginal and Torres Strait Islander peoples. VAHS HREC approval was required to obtain approval to use the ARRQ for research purposes.

## Conflicts of Interest

Erin Cunningham who led this study is a service manager of Pinangba. The other authors declare no conflicts of interest.

## Supporting information


**Data S1.** dar14095‐sup‐0001‐TableS1‐S2‐FigureS1.

## Data Availability

The data are not publicly available due to ethical restrictions.
