# Supplementary material for: Assessing the Processes and Experiences of Using and Implementing a Routine Data Collection System at Two Aboriginal Alcohol and Other Drug Residential Services Located in Rural Queensland
Source: Drug Alcohol Rev. 2025 Jun 8;44(5):1556–63. doi: 10.1111/dar.14095 (PMC12228033; doi:10.1111/dar.14095)
Supplement: Supplementary file 1 — Data S1. dar14095‐sup‐0001‐TableS1‐S2‐FigureS1. [file DAR-44-1556-s001.docx]

**Supporting Information**

**Experience and outcome survey details and outcomes**

The assessment of the family centric model was not the intention of this manuscript, but rather to focus on the feasibility and implementation of a data collection system that could be employed routinely by the services to measure client experience and client outcome and be integral to ongoing quality improvement initiative. We have however added the descriptive findings of the experience and outcome survey for additional context. Although ongoing data collection and analysis are necessary to validate these findings over time, initial results suggest promising outcomes in enhancing the health and wellbeing of their clients. It also shows positive results in terms of clients’ experiences with accessing and engaging with both Pinangba services. Continuous evaluation will ensure that Pinangba's services remain effective in delivering quality care and enhancing the outcomes of their clients.

**Additional information on methodology**

*Description of client outcome survey*

The client outcome survey comprises five sections. Section 1 collects demographic data. It includes items on marital status, usual accommodation, living arrangements, work situation, level of education, legal circumstances, referral type, diagnosis of mental health illness, medication use, Aboriginal and/or Torres Strait Islander status and sex. Section 2 assesses strengths and resources associated with resilience, healing and recovery among Aboriginal and Torres Strait Islander clients. The validated short version of the validated Aboriginal Resilience and Recovery Questionnaire (ARRQ) [1] was used. The short form is a 19 item questionnaire designed to assess a range of Aboriginal and Torres Strait Islander strengths and resources. Questions are answered using a five point Likert Scale response category from “not at all” to “a lot”. The ARRQ is grouped into three areas: cultural strengths and resources (11 items); relationship strengths and resources (4 items); and personal strengths and resources (4 items). The three scores can be combined to calculate a total strength score (scoring range: 19 to 95). This section is skipped for clients who do not identify as Aboriginal and/or Torres Strait Islander.

Section 3 collects data on the alcohol and other drug use of clients and is based on the following validated tools: the Severity of Dependence Scale [2], the Australian Alcohol Treatment Outcome Measure [3], and the Brief Treatment Outcome Measure-Concise [4]. It includes 20 items assessing what substance is causing the client the most concern in the last three months. It also assesses the number of days and the quantity used in the last four weeks. This section is only completed at admission and post treatment (as it is assumed that no alcohol and other drugs [AOD] is used while in treatment).

Section 4 includes 10 items measuring the confidence and capacity of clients to manage certain aspects of their life (e.g., having the confidence to find a safe place to live or the skills to secure employment). Clients and staff during the Phase 1 interviews identified it as an important area to measure. However, as no (validated) tools exists that capture this, the items were developed in consultation with clients, family members, staff, and Advisory Group members. Questions are answered using a five point Likert Scale response category from “not at all” to “a lot”. A “not applicable” option is also available (eg., not all clients have children in care). The higher a statement score is, the more confident a client feels in that skill (scoring range: 1 to 5).

Section 5 assesses quality of life within the context of an individual’s culture, value systems, personal goals, standards and concerns. The World Health Organization Quality of Life – BREF [5] was used to measure this. The WHOQOL-BREF consists of 26 items. Questions are answered using five-point Likert Scale response categories with answers from “very dissatisfied” to “very satisfied”, “not at all” to “an extreme amount”, and “never” to “always”. It is recommended that the first two items of the WHOQOL-BREF measuring overall quality of life and overall health are analysed separately [6]. The higher the score the more satisfied participants are with their overall quality of life or health (scoring range: 1 to 5). The other 24 items are used to produce four domain scores: a physical health domain (7 items); a psychological health domain (6 items); a social relationships domain (3 items); and an environment domain (8 items). Two additional items measure overall quality of life and overall health. The WHOQOL-BREF uses a five-point Likert scale with answers from “very dissatisfied” to “very satisfied”, “not at all” to “an extreme amount”, and “never” to “always”. Transformed domain scores resulted in a 0–100 scale, in which higher scores indicate higher quality of life. Section 6 is an open text box allowing clients to provide feedback. More details around the variables of the different sections can be found below.

*Analysis of outcome and experience survey*

Client experience survey: Demographic data were summarised using descriptive statistics. Considering the high levels of agreement with all statements (and to improve readability), only the “agree” and “strongly agree” responses are reported on. The total score for each statement was calculated. The higher the score, the more satisfied clients were. Response categories for the Likert scale items were collapsed (strongly disagree/ disagree/ slightly agree vs. agree/ strongly agree) for analysis. All analyses were conducted in SPSS Version 27.

Client outcome survey: Demographic data were summarised using descriptive statistics. The total strength score of the ARRQ was calculated. The higher the score, the more resilient participants are. The total scores of the confidence and capacity items were separately scored and plotted in a graph on group level across the five time points. The total scores of the WHOQOL-BREF were calculated for the two individual items and the four domains. A higher score means a higher quality of life. The overall scores for all sections were plotted in a graph on a group level for all five time points. No tests of association were conducted for the client outcome survey due to the small sample size (particularly for the collection time points after admission). We however feel it is important to report the findings of this pilot as little evaluation research exists on Aboriginal alcohol and other drugs treatment services or alcohol and other drugs services adopting a family-centric model of care.

**Findings experience and outcome survey**

*Client experience survey outcomes*

Most clients who filled out the client experience survey agreed or strongly agreed with all statements. See Table 1 for more detail regarding the different statements and their scoring across the three surveys.

**Table S1.** Comparison of self-reported experience of alcohol and other drug services received at Pinangba at three different timepoints: percentage ‘agreed’ and ‘strongly agreed’*

| Statements | Sep 22 (n=11) | Mar 23 (n=13) | Oct 23  (n=8) | *χ2* | *p* |
| --- | --- | --- | --- | --- | --- |
| S1: I have been supported to start doing things that I want to do | 100% | 100% | 75.0% | 6.400 | 0.056 |
| S2: I feel better about myself because of this program | 100% | 100% | 75.0% | 6.400 | 0.056 |
| S3: I understand myself better because of this program | 100% | 100% | 62.5% | 9.931 | 0.011 |
| S4: I have enough privacy here | 100% | 100% | 50% | 13.714 | 0.002 |
| S5: I am given enough space by other people in this program | 100% | 100% | 75.0% | 6.400 | 0.056 |
| S6: I understand better why I have used drugs and/or alcohol because of the program | 100% | 100% | 75.0% | 6.400 | 0.056 |
| S7: I have enough one-to-one sessions | 100% | 84.6% | 62.5% | 4.941 | 0.089 |
| S8: I am supported to look after my health, financial, and legal problems | 100% | 100% | 50.0% | 13.714 | 0.002 |
| S8: I can get help for any difficulties I have | 90.9% | 100% | 62.5% | 6.545 | 0.048 |
| S9: I know what the rules are and what will happen if I don’t follow the rules | 100% | 100% | 87.5% | 3.097 | 0.250 |
| S10: I think the rules make sense | 81.8% | 100% | 87.5% | 2.441 | 0.326 |
| S11: My day is structured here | 100% | 100% | 100% | - | - |
| S12: I am provided with a schedule so that I know what to do with my time | 100% | 100% | 87.5% | 3.097 | 0.250 |
| S13: I am provided with opportunities to exercise | 90.9% | 92.3% | 75.0% | 1.534 | 0.650 |
| S14: I have access to healthy food | 100% | 100% | 87.5% | 3.097 | 0.250 |
| S15: I think this place is clean and hygienic | 100% | 92.3% | 100% | 1.509 | 1.000 |
| S16: I feel supported and understood by other people in this program | 100% | 92.3% | 62.5% | 6.418 | 0.068 |
| S17: Staff treat me like a person and not an addict | 100% | 92.3% | 62.5% | 6.418 | 0.068 |
| S18: I am supported to focus on my recovery | 100% | 100% | 87.5% | 3.097 | 0.250 |
| S19: My family and friends have been provided with information about recovery | 100% | 76.9% | 62.5% | 4.544 | 0.101 |
| S20: I am able to cope more with my everyday life outside the program | 100% | 92.3% | 62.5% | 6.418 | 0.068 |
| S21: I have been linked up with other services to support me when I leave this program | 100% | 84.6% | 50.0% | 7.763 | 0.025 |
| S22: I can get information from staff about where else I can go for help | 100% | 92.3% | 75.0% | 3.480 | 0.238 |

* Values are reported as % of participants endorsing “agree” or “strongly agree”

When analysing the surveys by their respective timepoints, significant statistical differences were found between the three timepoints, notwithstanding the very small sample sizes.

Notably, clients for the third survey round seem to express wanting more support with other health and social issues (outside of their AOD use). For example, only 50% of clients agreed that they feel supported to look after their health, financial, and legal problems (statement 8). However, some of the lower percentage scores likely reflect the higher number of new clients that participated in the third survey, as these clients are still getting familiar with the program and are at the beginning of their treatment journey (e.g., I understand myself better because of this program).

Clients were also asked about what Pinangba is doing well and what could be improved. All comments were generally positive, describing the services as a comfortable place where everyone is friendly. There was also positive feedback around the program, specifically around the program teaching clients about themselves, spending more time with their kids, and keeping them on track. However, it was noted that communication between staff and clients could be strengthened, that more support could be given to address issues other than their alcohol and other drug use, and better preparation of what life looks life when entering the service.

*Client outcome survey outcomes*

Data are only shown for the three timepoints within treatment as almost all clients did not complete a survey post treatment. AOD use was only measured at admission and post treatment as clients are required to be abstinent while in treatment. We do not report on this outcome as there was no post treatment data.

Client characteristics

The client characteristics are based on the 26 clients who filled out an admission survey. In total, 92.3% identified as Aboriginal and/or Torres Strait Islander, 57.7% of participants were female, 69.2% were not diagnosed with a mental health disorder and most clients were self-referred (53.8%).

Alcohol was the primary drug of concern for most clients (76.9%). In relation to their primary drug of concern, 50% of clients often or (nearly) always felt that their AOD use was out of control, 26.9% of clients often or (nearly) always felt anxious or worried about missing the substance, 53.8% of clients often or (nearly) always felt worried about their AOD use, and 80.7% of clients often or (nearly) always wished that they could stop using.

For the clients who reported drinking in the last four weeks (n=13), clients on average consumed alcohol for 9.5 days in the last four weeks before admission (ranging between 2 and 30 days), with the average standard drinks for those days being 30.5 (ranging from 10 to 52 standard drinks a day). Only one person reported to have used cannabis for 1 day in the past four weeks before admission, and none of clients reported using amphetamines on any of the days for that period. See Table 2 for more information.

**Table S2.** Pinangba client characteristics based on admission surveys

|  | Admission survey (n=26) |
| --- | --- |
| Aboriginal and/or Torres Strait Islander (%) | 92.3% |
| Age (mean) | 37.3 years |
| Sex (%) | |
| Male | 42.3% |
| Female | 57.7% |
| Employment (%) | |
| Employed | 7.7% |
| Unemployed | 42.3% |
| Pension | 26.9% |
| Other | 23.0% |
| Mental health diagnosis (%) | |
| No | 69.2% |
| Yes | 30.8% |
| Legal circumstances (%) | |
| No outstanding legal matters | 69.2% |
| Probation or parole | 23.1% |
| Bailed (no pending court matters) | 7.7% |
| Primary drug of concern (%) | |
| Alcohol | 76.9% |
| Amphetamines | 15.4% |
| Cannabis | 7.7% |
| Referral type (%) | |
| Self | 53.8% |
| Other community or health care services | 15.4% |
| Mental health service | 7.7% |
| Hospital | 7.7% |
| Other (e.g., AOD service) | 15.3% |

The Aboriginal Resilience and Recovery Questionnaire (ARRQ)

On a group level, a small positive trend was found between admission and 2 months (within treatment). The total strength score of the ARRQ slightly increases (particularly at 2 months). Clients overall seem to strengthen their cultural, relationship and personal strengths and resources whilst in treatment. See Figure 1.

Confidence and capacity statements

In general, respondents rated feeling more confident in their skills over the duration of treatment, apart from statement 1 (finding a safe place to live) and 8 (feeling confident in saying no when AOD is offered). Particularly statements 2 (starting education or training), 3 (finding a job) and 10 (accessing support services) improved for clients while in treatment. The results of the ten statements have been split into two graphs to improve readability of the total scores. See Figure 2 and 3.

WHOQOL-BREF

It shows that clients rate their overall quality of life (Q1) and overall health (Q2) as high at admission, and it slightly improves while in treatment. It however needs to be noted that the sample size is lower at 2 months and discharge, making it hard to compare total scores. See Figure 4.

A positive trend can also be seen on a group level for the first three domains (Domain 1: Physical health; Domain 2: psychological health; Domain 3: social relationships). Domain 4 (environment) seems to stay much the same over time. See Figure 5.

**Figure S1.** Aboriginal Resilience and Recovery Questionnaire total score on a group level (scoring range: 19 to 95)

**Figure S2.** Average of each confidence and capacity statements (scoring range: 1–5) on a group level for statement 1–5

**Figure S3.** Average of each confidence and capacity statements (scoring range: 1–5) on a group level for statement 6–10

**Figure S4.** Overall quality of life (QoL) and overall health total score (scoring 1–5) on a group level

**Figure S5.** Domain total score (scoring 0–100) on a group level

**References**

1. Gee G, Sheridan S, Charles L, Dayne L, Joyce L, Stevens J, et al. The Her Tribe and His Tribe Aboriginal-Designed Empowerment Programs. International Journal of Environmental Research and Public Health. 2022;19(4).

2. Gossop M, Best D, Marsden J, Strang J. Test-retest reliability of the Severity of Dependence Scale. Addiction. 1997;92(3):353-.

3. Simpson M, Lawrinson P, Copeland J, Gates P. The Alcohol Treatment Outcome Measure (ATOM): a new clinical tool for standardising outcome measurement for alcohol treatment. Addict Behav. 2009;34(1):121-4.

4. Lawrinson P, Copeland J, Indig D. The Brief Treatment Outcome Measure: Opioid Maintenance Pharmacotherapy. Sydney, Australia: National Drug and Alcohol Research Centre, University of New South Wales; 2003.

5. The WHOQOL Group. Development of the World Health Organization WHOQOL-BREF quality of life assessment. The WHOQOL Group. Psychol Med. 1998;28(3):551-8.

6. World Health Organization. WHOQOL-BREF : introduction, administration, scoring and generic version of the assessment : field trial version. Geneva: World Health Organization (WHO); 1996.
